# Infectious bronchitis virus from chickens in Al-Hasa, Saudi Arabia 2015-2016

**DOI:** 10.14202/vetworld.2019.424-433

**Published:** 2019-03-19

**Authors:** Musaed Abdulaziz Alsultan, Mohamed Ali Alhammadi, Maged Gomaa Hemida

**Affiliations:** 1 Training Veterinary Center, Ministry of Environment, Water and Agriculture, Saudi Arabia; 2 Department of Microbiology and Parasitology, College of Veterinary Medicine, King Faisal University, Saudi Arabia; 3 Department of Virology, Faculty of Veterinary Medicine, Kafrelsheikh University, Egypt

**Keywords:** Al-Hasa, molecular, infectious bronchitis, isolation, molecular, phylogenetic

## Abstract

**Aim:**

This study aimed to isolate some of the currently circulating infectious bronchitis virus (IBV) strains from some broiler chicken farms in Al-Hasa and to do some molecular characteristics of these strains.

**Materials and Methods:**

We collected 300 tissue specimens, including the trachea, bronchi, lungs, and kidneys from some four commercial chicken farms showing respiratory manifestations. We tested these tissue specimens by the real-time polymerase chain reaction (RT-PCR) and gel-based PCR. We selected some PCR positive samples for isolation in the embryonated chicken eggs (ECE). We sequenced some PCR-positive samples and conducted phylogenetic analysis based on the obtained sequences.

**Results:**

Our molecular surveillance revealed that 31.6% of the tested specimens were IBV positive by PCR. We selected some positive specimens showing low Ct values by the qRT-PCR for virus isolation by the ECE. The infected eggs showed hemorrhage, dwarfing, and death in some cases after three passages in the ECE. We sequenced some of the positive PCR specimens and used the obtained sequences to draw the phylogenetic tree based on the partial IBV-ORF-1a, N, and S1 gene sequences. The phylogenetic trees based on the IBV-N and S1 gene sequences showed that the circulating IBV strains in Al-Hasa during 2016 was showing a high degree of identity to some strains from Taiwan and Italy. Meanwhile, the grouping of these strains based on the IBV-S1 sequences revealed that the currently circulating IBV strains in Al-Hasa belonged to Gr.I.7 along with strains from Taiwan.

**Conclusion:**

Our results confirmed the continuous circulation of the IBV among the chicken population in Al-Hasa despite the intensive application of vaccines against this virus.

## Introduction

Infectious bronchitis virus (IBV) is a highly contagious respiratory viral disease of chickens of all ages. The IBV belongs to the order *Nidovirales*, family *Coronaviridae*, and genus Gammacoronavirus [1,2]. The IBV genome is a positive sense RNA molecule with a high tendency to frequent changes and recombination. There is a high degree of genetic diversity among IBV strains. Until recently, there was no available system for the definite grouping and identification of various IBV genotypes. One recent study used the combination between the phylogenetic analysis and the pairwise similarities on both the nucleotides and the amino acid levels to do fine mapping of the IBV genotypes [3]. This study used the full-length S1 gene sequences to develop a novel classification system for the IBV genotypes and lineages [3]. Based on the comparison of the coding sequence of the S1 gene from 1652 IBV strains, they were able to categorize the currently circulating IBV strains into 6 genotypes and 32 lineages [3]. The IBV infection usually causes high economic losses among the poultry industry. It is quite possible to become endemic in the chicken industry in some regions of the world. The virus has wide tissue tropism, including the respiratory, digestive, renal, and reproductive systems of the affected birds. It may affect the oviduct and lead to low production and low-quality eggs or may cause severe renal complications and mortality among the affected birds [4]. The IBV-infected birds usually shed the virus in their body secretions such as respiratory and the gastrointestinal tract secretions. These birds may remain active shedders of the virus for up to several weeks post-infection [5]. Secondary bacterial infections (*Escherichia coli* and *Mycoplasma gallisepticum*) always exaggerate the viral pathogenesis and auscultate the mortality rates among the infected chicken population [6]. The IBV infections continue to be a major problem to the poultry industry worldwide. In spite of the availability of several IBV vaccines, the virus continues to cause many outbreaks among the chicken farms in both broiler and layer settings [7].

Efforts to control spreading of the IBV infections through vaccination resulted in some variable outcomes. Many IBV strains and serotypes have emerged since its discovery >80 years ago; meanwhile, the misuse of the IBV vaccines complicates the evolution and emergence of new IBV strains [8]. One possible explanation for the emergence of new IBV strains is the poor proofreading capability of the viral RNA polymerase. This resulted in high mutation rates alongside the viral genome. This leads to the emergence of new IBV strains occasionally [9,10]. Many IBV vaccines are commercially available, including inactivated, live attenuated, and recombinant. The live-attenuated vaccines are the most commonly used in all types of poultry; these provide a good immune response; however, there is a possibility of revert to virulence. Meanwhile, the inactivated vaccines are usually administered to layers and breeder’s chickens before the laying time as booster vaccines [11,12].

The IBV infection in chicken was first reported in Saudi Arabia in 1984 in field samples by the real-time polymerase chain reaction (RT-PCR) using N primers [13]. Another study reported the circulation of the IBV/4/91 serotype in Saudi Arabia in 2000 using the partial S gene [14]. Several IBV genotypes were previously reported in Saudi Arabia, including CH/CK/LDL/971, IS/720/99, ISVariant2/98, and the D24 [15]. We recently reported the circulation of very virulent IBV strains in chicken farms from Eastern Saudi Arabia [16].

Several IBV variants and genotypes are currently circulating in the Middle East, Asia, and North Africa such as Iraq, Egypt, Libya, Iran, and Jordan [17]. Furthermore, many outbreaks were reported in Saudi Arabia, especially in the central region of the country [15,18,19]. However, the full molecular characterization of these IBV strains and variants is not well reported yet.

This study aimed for isolation and molecular characterization of the IBV circulating strains of the IBV in Al-Hasa in the eastern region of Saudi Arabia. This region is one of the major hubs for intensive poultry production in Saudi Arabia.

## Materials and Methods

### Ethical approval

We conducted this study according to the King Faisal’s University Animal Ethics protocols and the National Committee of Bio-Ethics, King Abdul-Aziz City of Science and Technology, Royal Decree No. M/59 (http://www.kfsh.med.sa/KFSH_WebSite/usersuploadedfiles%5CNCBE%20Regulations%20ENGLISH.pdf). The Animal Ethics Committee of the King Faisal’s University approved this protocol.

### Sample collection and processing

We conducted molecular surveillance for IBV on some chicken farms across Al-Hasa from November 2015 to April 2016. Birds in these farms were suffering from acute respiratory signs and high morbidity and mortality rates. These outbreaks were mapped around three major cities in the Al-Hasa province (Table-1), Saudi Arabia (Al-Hufuf, Mahasen, and Almubarez). 100 tissue specimens (trachea, lung, and kidney) representing four suspected IBV outbreaks were collected. These samples were stored at −80°C for further processing. Briefly, 1 g per each tissue specimens was ground in a sterile mortar mixed with 9 ml of phosphate-buffered saline (PBS) and sterile sands. Tissues were then centrifuged at 5000 rpm for 15 min. The supernatants were harvested and stored at −80°C until use.

**Table-1:** Summary of the collected tissue specimens and their geographical distribution 2015-2016.

No	Type	Date of collection	Age/days	Signs	Number of chicken	Organs	Farm No.
1	B	10/2015	14-21	RS	40	Lu, T, K	4
2	B	11/2015	14-21	RS	15	Lu, T, K	10
3	L	2/2016	14-21	RS	20	Lu, T, K	8
4	L	4/2016	14-21	RS	25	Lu, T, K	9
Total					100	300	

B=Broiler, L=Layer, RS=Respiratory signs, Lu=Lung, T=Trachea, K=Kidney

### Isolation of IBV through embryonated chicken eggs (ECE)

Isolation of the currently circulating IBV strains from some poultry farms in Al-Hasa was carried out by the inoculation of the ECE. We used 9-11 days’ chicken embryos from native breeds (non-IBV vaccinated) and proved to be IBV-negative antibodies by ELISA. The inoculum was prepared as follows; IBV-suspected tissue suspensions were centrifuged for 10 min at 5000 rpm. Antibiotic mixture was added (penicillin and streptomycin) to each tissue suspension. We used 100 µl per each tissue suspension to inoculate through allantoic route into five embryonated eggs [20]. The inoculated eggs were incubated at 37°C. Negative control PBS-inoculated eggs were done in parallel to the experimental infection of the ECE. We observed the inoculated eggs daily by candling for or up to 3 days post-inoculation. Any early deaths within 24 h post-inoculations were excluded due to the non-specific trauma-related death [21].

### Viral RNA extraction

The total viral RNAs were extracted from tissue using the QIAamp Viral RNA Mini Kit (Qiagen cat. NO. 52906 Qiagen, Germany] as per the manufacturer’s recommendations. The eluted viral RNAs were stored at −80°C for further testing. The positive control (the commercial live IBV-H120 vaccine for IBV, Veterinary Vaccine Production Centre, KSA) was processed in parallel to each batch of specimens as described above.

### The qRT-PCR

The RT-PCR was performed using the IBV RT commercial kits (Subang Jaya, Selangor DE, Malaysia) (qPCR/RT-Qpcr kits Kestrel BioScience LLC, Doc.No. cat# AV1007011). The reactions were conducted as per the kit’s instructions with some minor modifications. Briefly, each reaction consisted of 2×RT-PCR master mix (10 µl, IBV PPM 1 µl, PPM IEC 1 µl, nuclease-free water 3 µl, and 5 µl of RNA template) in a total reaction volume of 20 µl. Meanwhile, we used 5 µl of nuclease-free water as negative control. Furthermore, a positive control reaction was carried out in parallel to each run. Duplicate reactions were done per each specimen. The cycling parameters were 55°C for 10 min, then 95°C for 8 min, followed by 50 cycles at 95°C for 10 s, and 60°C for 1 min.

### Oligonucleotides

We designed some primers to amplify the partial IBV-N gene. We designed the IBV-N primers based on the IBV-N (accession no: KT762154). The nucleotide sequences of the used IBV-N gene are IBV-NF (5’-CGCTGGAGAATTTCCTCTTG-3’) and the IBV-NR (5’-CTAGTCCCTAGCAGCCATGC-3’). Meanwhile, we used the following primers, IBV-S1F (5’-ACCGGCTGATGGATGGCAT-3’) and the IBV-S1 (5’-TTGCTTACAACCACCCTGTAG-3’), to amplify the partial IBV-S1 gene.

### The reverse-transcriptase polymerase chain reaction (RT-PCR)

The RT-PCR reactions were conducted in a 25 µl reaction volumes using the one-step RT-PCR kit (Qiagen, Valencia, CA NO. 210212). The reactions were conducted as per the manufacturer’s instructions. The reaction mixture consists of 10 µl of 5×reaction buffer, 2 µl of each primer, 2 µl of enzyme mixture, 2 µl of dNTP mixture (400 µM of each dNTP), 3 µl of the RNA template, and 4 µl of RNase-free water. Reverse transcription was started at 50°C for 30 min; initial Taq polymerase activation was performed at 95°C for 15 min, along with 40 cycles of denaturation at 94°C for 1 min, annealing at 55°C for 1 min, and then extensions at 70°C for 1 and 10 min.

### Agarose gel electrophoresis

For visualization of the amplified PCR products, electrophoresis of 10 µl of each PCR products on 1% agarose gel was performed. This was done under the ultraviolet light and photographed by the Bio-Rad gel documentation system. 10 µl of per each PCR product was mixed with 3 µl of the loading buffer (blue/orange loading dye, Roche). A 100-bp, ready-to-load DNA-marker ladder was loaded in a separate, parallel lane.

### Next-generation sequencing

We processed a confirmed PCR-ECE-P3-IBV isolate for sequencing by the Illumina next-generation sequencing approach. We used this approach to decode the partial-length genome sequences of this IBV isolate as previously described [22].

### Phylogenetic analysis

We constructed the phylogenetic tree based on the obtained partial IBV-ORF1-a, IBV-N, and S1 gene sequences. We constructed the tree using the multiple alignments of these sequences with other IBV sequences retrieved from the GenBank done using the Mega-7 package, and phylogenetic analysis was done by the maximum likelihood method using the best-fit model determination and at least 500 bootstrap replicates as previously described [23]. Meanwhile, we used the Mega-7 software to calculate the pairwise distances between our IBV-S1 sequence and the available sequences from the GenBank.

### Statistical analysis

We applied the non-probability sampling strategy for our specimen collection with incidental assignment approach as previously described [24].

## Results

### Clinical signs and postmortem lesions of IBV-infected chickens in Al-Hasa, 2015-2016

**Figure-1:**
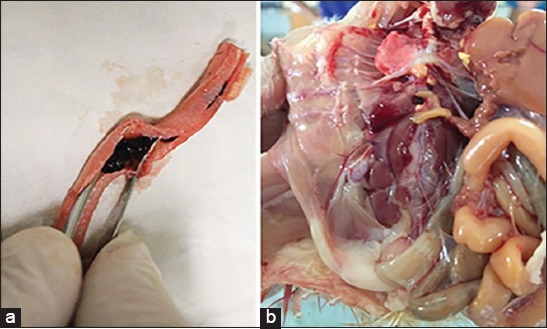
Postmortem lesions of the infectious bronchitis virus (IBV)-infected chickens in Al-Hasa region 2015-2016. (a) PM lesions of the IBV-infected chicken trachea showing congestion and hemorrhage of the tracheal mucosa and presence of hemorrhagic plug at the tracheal lumen. (b) Kidney of infected chicken infected with IBV showing congestion, swollen, and distinct lobos on the necropsy examination.

The IBV infection in chickens produces a wide range of clinical syndromes. Some birds showed respiratory manifestation in terms of coughing, sneezing, and nasal discharges. Necropsy examination of some IBV-infected birds revealed serous, catarrhal, or caseous exudate in the tracheal, nasal passage, sinus, and bronchi congestion (Figure-1a). The edema of the lungs, cloudy or fibrinous inflammation of the air sacs, occasionally contains a yellow caseous exudate. Edema and swelling of the kidneys were observed in some birds (Figure-1b).

### Molecular surveillance of IBV among some chicken farms in Al-Hasa 2015-2016

We conducted molecular surveillance to check the prevalence of IBV across the Al-Hasa from October 2015 to April 2016. We investigated four IBV outbreaks across the Al-Hasa province by the RT-PCR (Table-1). We tested 100 chickens’ specimens from non-vaccinated chickens using tissue specimens from the trachea, lungs, and kidneys by the RT-PCR technique. Our overall results showed that 19 pooled chickens’ tissue specimens were positive of 60 tested samples (31.6%). Simply, specimens from farm No: 8 is showing that 6 out of 12 specimens (50%) were positive. In the case of farm No: 4, 8 of 24 (33.33%) were IBV positive. However, three specimens of nine tested from one particular chicken farm were positive in case of farm No: 10. Interestingly, only two specimens from poultry farm on the farm No: 9 were positive of 15 tested specimens (13.33%) (Table-2).

**Table-2:** Summary of the results PCR tested sample tissues from chickens across Al-Hasa 2014-2016.

Farm No.	Number of chicken	Organs No.	Pooling	+Ve	−Ve	% (+Ve)
4	40	120	24	8	16	33.33
10	15	45	9	3	6	33.33
8	20	60	12	6	6	50
9	25	75	15	2	13	13.33
Total	100	300	60	19	41	31.67

PCR: Polymerase chain reaction

We already tested different tissue specimens, including the trachea, lungs, and kidneys, from some suspected IBV-infected birds. Our results are showing that 11 tracheal tissue specimens of 20 were positive, while 6 lung specimens of 20 were positive; meanwhile, only two specimens from the kidneys were positive (Table-3).

**Table-3:** Summary of the RT-PCR testing of suspected IBV tissue specimens from farms around Al-Hasa region 2014-2016.

Type of specimen	No	(+Ve)	(−Ve)	% (+Ve)
T	100	11	9	55
Lu	100	6	14	30
K	100	2	18	10
Total	300	19	41	31.67

No=number of birds, Lu=Lung, T=Trachea, K=Kidney, IBV=Infectious bronchitis virus, RT-PCR=Real-time polymerase chain reaction

### Isolation of IBV strains from some chicken farms in Al-Hasa 2015-2016

We processed 15 specimens (trachea, lungs, and kidneys) after pooling from three IBV outbreaks. We did the propagation for three passages. The IBV isolation was successful only in case of two specimens. The first specimen was collected from an RT-PCR-IBV-positive chicken trachea collected from farm no. 7. The second specimen was collected from positive IBV-RT-PCR lungs collected from chicken farm no. 5 area (Table-4). However, isolation from the inoculated kidney tissues from farm no. 3 was not successful (Table-4). The IBV infection of the ECE resulted in many pathological changes on the inoculated embryos such as dwarfing, congestion, hemorrhage, and death at different time points. No pathological changes, alteration, or death was reported in the negative control group of embryonated eggs in the three subsequent passages.

**Table-4:** Summary of the isolation of IBV from a field outbreak in some chicken farms in and around Al-Hasa region.

No.	Farm No.	Specimen	Number	Pooling	P1		P2		P3	
		
					+Ve	−Ve	+Ve	−Ve	+Ve	−Ve
1	7	T	15	3	2	1	2	1	2	1
2	5	Lu	15	3	1	2	1	2	1	2
3	3	K	15	3	-	3	-	3	-	3
Total			45	9	3	6	4	5	4	5

IBV=Infectious bronchitis virus

We used three tissue suspensions (trachea, lungs, and kidneys) from three specimens representing three different IBV outbreaks in some chicken farms from Al-Hasa region. We followed up these three inoculums on the ECE for three subsequent passages. The IBV-infected tracheal specimen caused death and induced some pathological changes in two embryos from the inoculated embryos in the three passages. However, one of them showed no pathological changes or death at the end of the experiment. Meanwhile, the IBV-infected lung tissue specimens showed that only one embryo was affected per each passage.

### Confirmation of the identity of the IBV-infected specimens by the RT-PCR

**Figure-2:**
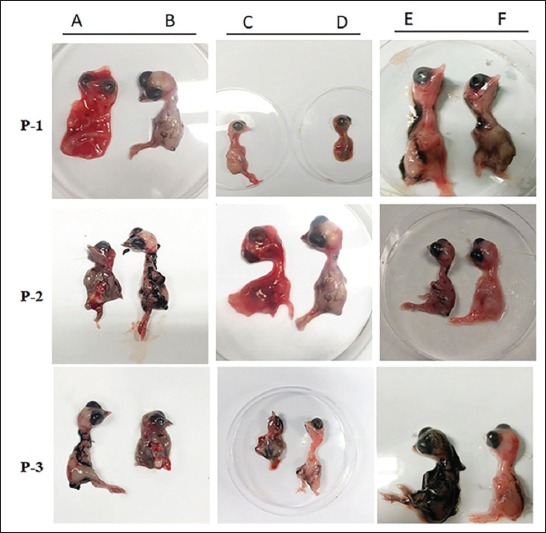
Isolation of the infectious bronchitis virus (IBV) strains using the embryonated chicken eggs. Isolation and propagation of the circulating IBV strains from chicken farms in Al-Hasa chicken farms 2015-2016 using ECE in three subsequent passages (P1-P3). The 10 days old, ECE inoculated with some selected IBV tissue suspensions trachea (c), lung (a), and kidney (f). Panels A and C are showing some pathological changes in the IBV-inoculated embryos (congestion, hemorrhage, malformation, and underdeveloped feathers). Panel F (kidney tissue suspensions) is showing that none of the inoculated embryos had any pathological changes. Panels B, D, and E are the sham phosphate-buffered saline negative control embryos showing normal size and no pathological changes.

We used commercial available RT-PCR kits to confirm the identity of some suspected IBV-infected chicken tissues, as well as some fluids from the embryonated egg passages. We carried out the RT-PCR amplification for three representative specimens from the ECE passages P-1-3. Meanwhile, we already included six original specimens from the IBV-suspected outbreaks from different farms, including some pooled tissue specimens (trachea, lungs, and kidneys). Furthermore, we already included the IBV vaccine as a positive control of the reaction in addition to the kits positive control in the reactions. In addition to a negative control used a non-DNA template. Our results show that 6/9 (66.67%) of the three tested specimens representing embryonated eggs passages 1-3 were positive. Three specimens from selected IBV specimens were positive, as shown in Figure-2.

### Phylogenetic analysis of the currently circulating IBV strains in Al-Hasa regions 2015-2016

**Figure-3:**
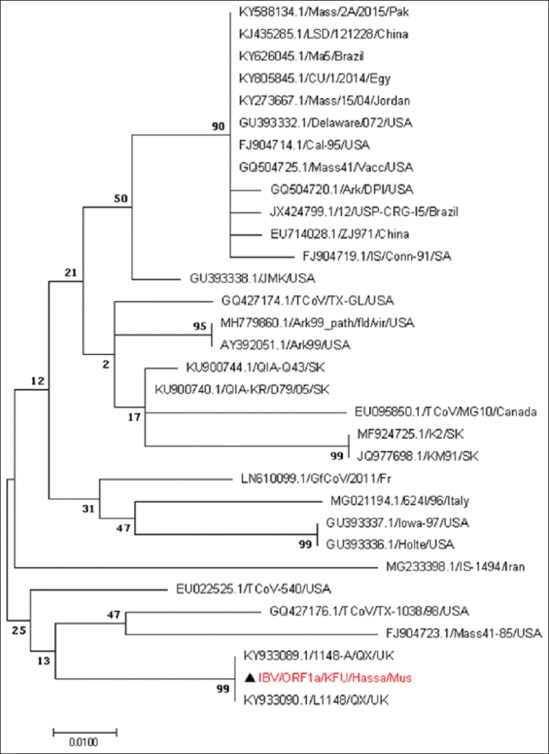
Phylogenetic analysis based on the partial ORF-1a gene for the circulating infectious bronchitis virus (IBV) strains from some chicken farms in Al-Hasa region 2015-2016. Phylogenetic tree of the obtained partial Saudi IBV-ORF-1a gene isolated from Al-Hasa region 2015-2016. The maximum likelihood phylogenetic tree based on the partial IBV-ORF-1a gene. The bootstrap is 1000. The reported local IBV sequences clustered together with other QX-IBV strains from the UK. A red triangle marks the reported sequence in this study.

**Figure-4:**
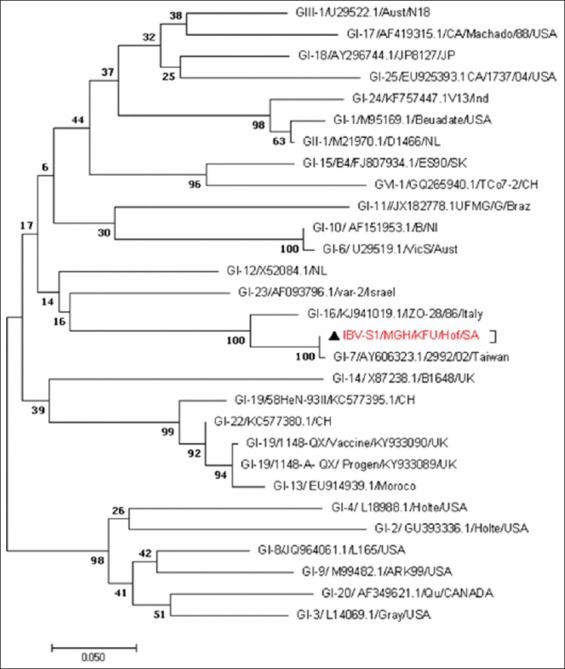
Phylogenetic analysis based on the partial S1 gene for the circulating infectious bronchitis virus (IBV) strains from some chicken farms in Al-Hasa region 2015-2016. Phylogenetic tree of the obtained partial Saudi IBV-S1 gene isolated from Al-Hasa region 2015-2016. The maximum likelihood phylogenetic tree was based on the partial IBV-S1 gene. The bootstrap is 1000. The reported local IBV sequences clustered together with other IBV strains from Italy and Taiwan. A red triangle marks the reported sequence in this study.

**Figure-5:**
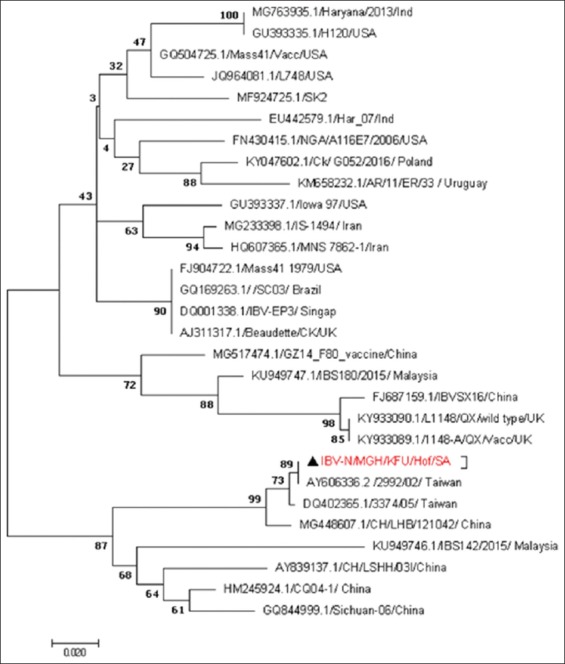
Phylogenetic analysis based on the partial N gene for the circulating infectious bronchitis virus (IBV) strains from some chicken farms in Al-Hasa region 2015-2016. Phylogenetic tree of the obtained partial Saudi IBV-S1 gene isolated from Al-Hasa region 2015-2016. The maximum likelihood phylogenetic tree was based on the partial IBV-N gene. The bootstrap is 1000. The reported local IBV sequences clustered together with other IBV strains from Taiwan and China. A red triangle marks the reported sequence in this study.

Based on the reported IBV-ORF1a sequences, these strains were closely related to IBV-L1148 strains (Figure-3). The phylogenetic tree based on the partial IBV-S1 genes revealed that these strains were closely related to other IBV strains from Taiwan and Italy (Figure-4). The evolutionary relationship analysis based on the IBV-S1 sequences confirmed the S1-based analysis (Figure-4). The phylogenetic analysis based on the generated IBV-N gene sequences clearly showed that the circulating strains were closely related to the IBV strain reported from Taiwan and China (Figure-5).

Both the pairwise distance (Table-5) and the phylogenetic analysis based on the generated IBV-S1 sequences of our isolates confirmed the blast results (Figures-4 and 5).

**Table-5:** Pairwise distance analysis of partial S1 gene for the circulating IBV strains in some chicken farms in Al-Hasa region 2015–2016.

GI.12/NL/X52084	0.22	0.22	0.20																										
GI.23/Isra/AF093796	0.21	0.22	0.21	0.19																									
GI.19/QX/_Pro/KY933089	0.28	0.28	0.06	0.23	0.23																								
GI.19/QX/Vacc/KY933090	0.28	0.29	0.06	0.23	0.24	0.00																							
GI.16/It/KJ941019	0.07	0.07	0.24	0.22	0.23	0.25	0.26																						
GI.15/Kor/B4/FJ807934	0.27	0.26	0.28	0.23	0.19	0.27	0.27	0.26																					
GVI.1/CH/GQ265940	0.26	0.27	0.26	0.26	0.25	0.30	0.30	0.26	0.14																				
GI.22/CH/KC577380	0.26	0.26	0.04	0.20	0.21	0.02	0.02	0.24	0.25	0.27																			
GIII.1/Aust/U29522	0.26	0.25	0.23	0.21	0.22	0.24	0.25	0.25	0.19	0.22	0.22																		
GI.11/Braz/JX182778	0.26	0.26	0.26	0.25	0.26	0.27	0.27	0.25	0.26	0.29	0.25	0.27																	
GI.24/V13/Ind/KF757447	0.30	0.30	0.27	0.23	0.27	0.31	0.31	0.25	0.20	0.23	0.28	0.19	0.25																
GI.1/Beu/USA/M95169	0.30	0.30	0.28	0.21	0.27	0.29	0.29	0.26	0.20	0.22	0.27	0.17	0.26	0.08															
GI.18/JP/AY296744	0.28	0.29	0.25	0.24	0.19	0.28	0.29	0.25	0.22	0.21	0.27	0.13	0.27	0.19	0.19														
GI.21/Spain/DQ064806	0.27	0.28	0.25	0.28	0.21	0.21	0.22	0.24	0.27	0.26	0.21	0.27	0.28	0.36	0.35	0.27													
GI.25/CA/USA/EU925393	0.29	0.29	0.24	0.23	0.22	0.25	0.26	0.24	0.26	0.26	0.23	0.18	0.26	0.20	0.19	0.15	0.29												
GI.8/L165/USA/JQ96406	0.27	0.28	0.24	0.26	0.23	0.24	0.25	0.24	0.28	0.32	0.22	0.29	0.25	0.25	0.26	0.25	0.25	0.24											
GI.10/B/Nl/AF151953	0.30	0.29	0.27	0.23	0.23	0.27	0.28	0.28	0.23	0.28	0.26	0.18	0.22	0.27	0.24	0.25	0.24	0.27	0.29										
GI.20/CAN/AF349621	0.31	0.31	0.28	0.30	0.25	0.29	0.29	0.28	0.27	0.31	0.27	0.31	0.26	0.25	0.27	0.26	0.30	0.27	0.15	0.31									
GI.14/Bel/X87238	0.30	0.31	0.23	0.23	0.27	0.24	0.24	0.29	0.28	0.29	0.23	0.28	0.28	0.31	0.34	0.26	0.14	0.30	0.25	0.25	0.30								
GI.6/VicS/U29519	0.31	0.30	0.28	0.24	0.23	0.28	0.28	0.28	0.23	0.28	0.26	0.19	0.22	0.26	0.23	0.25	0.24	0.27	0.28	0.00	0.30	0.25							
GI.3/Gray/USA/L14069	0.32	0.32	0.25	0.29	0.24	0.25	0.26	0.28	0.29	0.33	0.23	0.30	0.23	0.29	0.31	0.28	0.27	0.30	0.14	0.27	0.15	0.28	0.26						
GI.4/Holte/USA/L18988	0.33	0.32	0.28	0.28	0.27	0.28	0.28	0.27	0.29	0.36	0.27	0.28	0.29	0.30	0.32	0.28	0.28	0.28	0.18	0.31	0.22	0.31	0.32	0.20					
GI.9/USA/M99482	0.29	0.29	0.20	0.28	0.24	0.22	0.22	0.26	0.27	0.29	0.21	0.28	0.25	0.26	0.27	0.25	0.28	0.28	0.13	0.28	0.17	0.34	0.28	0.14	0.18				
GI.2/USA/GU393336	0.34	0.33	0.32	0.32	0.29	0.31	0.32	0.31	0.29	0.37	0.29	0.33	0.32	0.31	0.32	0.33	0.33	0.32	0.18	0.31	0.21	0.33	0.31	0.19	0.23	0.25			
GI.17/USA/AF419315	0.33	0.33	0.27	0.28	0.23	0.25	0.25	0.30	0.27	0.26	0.23	0.14	0.30	0.27	0.23	0.16	0.27	0.21	0.28	0.21	0.37	0.30	0.22	0.31	0.33	0.29	0.34		
GII.1/NL/M21970	0.30	0.29	0.26	0.20	0.24	0.27	0.27	0.23	0.17	0.21	0.25	0.17	0.25	0.06	0.02	0.18	0.34	0.19	0.24	0.24	0.24	0.32	0.23	0.28	0.29	0.25	0.29	0.23	
GI.13/Moroc/EU914939	0.31	0.32	0.08	0.25	0.25	0.02	0.02	0.28	0.27	0.31	0.04	0.25	0.27	0.31	0.29	0.29	0.22	0.26	0.23	0.26	0.29	0.23	0.27	0.24	0.27	0.22	0.30	0.25	0.27

IBV=Infectious bronchitis virus

## Discussion

The geographical distribution of the suspected outbreaks represents different regions across the Al-Hasa. Some chicken farms were selected around the Al-Hasa region as targets for our study. Some of these farms were reporting IBV outbreaks based on the obvious clinical signs and post-mortem lesions, while birds in some others were apparently healthy. The clinical examination of the affected birds showed depression, ruffled feathers, loss of the body weight, respiratory rales, nasal discharge, and lachrymal discharge (data not shown). Necropsy examination of some affected birds revealed congested trachea, caseous and bloody plugs at the tracheal bifurcations (Figure-1a), congested lungs, and swollen kidneys (Figure-1b) in some cases. These clinical signs are very much typical of the IBV pattern of infection in poultry farms [25]. We conducted molecular surveillance for IBV among some IBV chicken outbreaks in Al-Hasa region 2014-2016. We selected some tissue specimens to be tested by the commercial IBV-RT PCR kits. Our results are showing that six specimens of nine (66.67%) were IBV positive (Table-4). We also selected some of these positive IBV specimens for the propagation and isolation of the currently circulating strains by the ECE. We assessed the success of the IBV isolation in two different ways. First, the inoculated embryos showed pathological changes relevant to the standard IBV inoculation in the ECE. These changes were in the form of congestion, dwarfing, hemorrhage, and death of the inoculated embryos. Second, we were able to detect the IBV signal by doing RT-PCR assays on the ECE tissues and fluids from different egg passages (data not shown). Furthermore, we confirmed the propagation of the IBV on the ECEs by another method using the truncated IBV-N primers through the RT-PR technique.

We conducted an IBV molecular surveillance among some selected chicken farms in Al-Hasa region by the RT-PCR technique using the conserved IBV-N primers. We tested 100 specimens from layers, and broiler non-vaccinated chicken’s flocks represented four suspected IBV outbreaks across the Al-Hasa. These tissue specimens were collected from various organs of the affected birds such as the trachea, lungs, and kidneys. We did pooling five organs per each tube considered as one specimen. We found that 19/60 specimens (31.67%) were positive (Table-4). Our results also showed that 11/20 (55%) of the tested trachea were positive, while 6/20 (30%) lungs were positive. However, 2/20 (10%) tested kidneys were IBV positive (Table-4).

We used the generated sequences of three independent genes (ORF1a, S1, and N) in the IBV genome to do the phylogenetic analysis of the circulating strains in Al-Hasa region during 2016. We found great overlapping and consistency of the phylogenetic analysis based on the IBV-S1 and N genes. The reported sequences were closely related to other IBV strains reported in Taiwan, Italy, and China (Figures-3-5). This high identity to foreign IBV strains may be due to that Saudi Arabia does import chickens from different countries including UK, China, Italy, and Taiwan. However, the IBV-ORF1a sequences revealed that these strains were related to other IBV strains belonging to the IBV-QX strain (L1148) (Figure-3). It is well known that there is a great diversity of S1 gene among IBV strains, which make it a strong candidate for the IBV classification [26]. Furthermore, novel variations were reported among most of the IBV-N gene [27,28]. This is suggesting the potential use of some IBV-S1 and maybe IBV-N genes for the classification of different IBV strains as described [3].

Our results confirmed recent reports about the circulation of different IBV strains in the kingdom and the eastern region in specific [15,29]. This indicates a wide distribution of IBV in the Al-Hasa in the Kingdom in general. In spite of the massive application of IBV vaccines in chicken farms under study, IBV is circulating, and thus, the currently used IBV vaccines under field conditions did not provide enough protection against the IBV infection. Another challenge in the context of this vaccination failure is the possibility of circulating of novel IBV strains that do not share any antigenic relationship with the currently used vaccines, and thus, protection cannot be achieved. We believe that IBV will continue to hit several chicken farms in the region and many other parts in the world. Further studies are highly recommended to do thorough molecular characterization of the currently circulating and the possible emerging IBV strains; thus, protection was usually achieved through the preparation of the homologous relevant specific IBV vaccines.

## Conclusion

IBV continues to pose a great risk to the poultry industry in the Al-Hasa, Saudi Arabia. There is continuous circulation of the IBV between the broiler and layer chicken in this area. The currently circulating strains of the IBV belong to the Gr.I-17 along with other genotypes from Taiwan, China, and Italy.

## Authors’ Contributions

MGH designed and performed the experiments, oversee the entire work, and performed data analysis and interpretation. MuAA conducted the fieldwork, performed some laboratory experiments, and drafted the manuscript. MoAA helped in the data analysis and reading of the manuscript. All authors read and approved the final version of the manuscript.
